# Regression based predictor for p53 transactivation

**DOI:** 10.1186/1471-2105-10-215

**Published:** 2009-07-14

**Authors:** Sivakumar Gowrisankar, Anil G Jegga

**Affiliations:** 1 Division of Biomedical Informatics, Cincinnati Children's Hospital Medical Center, Cincinnati, USA; 2 Department of Biomedical Engineering, University of Cincinnati, Cincinnati, USA; 3 Department of Pediatrics, University of Cincinnati College of Medicine, Cincinnati, USA

## Abstract

**Background:**

The p53 protein is a master regulator that controls the transcription of many genes in various pathways in response to a variety of stress signals. The extent of this regulation depends in part on the binding affinity of p53 to its response elements (REs). Traditional profile scores for p53 based on position weight matrices (PWM) are only a weak indicator of binding affinity because the level of binding also depends on various other factors such as interaction between the nucleotides and, in case of p53-REs, the extent of the spacer between the dimers.

**Results:**

In the current study we introduce a novel *in-silico *predictor for p53-RE transactivation capability based on a combination of multidimensional scaling and multinomial logistic regression. Experimentally validated known p53-REs along with their transactivation capabilities are used for training. Through cross-validation studies we show that our method outperforms other existing methods. To demonstrate the utility of this method we (a) rank putative p53-REs of target genes and target microRNAs based on the predicted transactivation capability and (b) study the implication of polymorphisms overlapping p53-RE on its transactivation capability.

**Conclusion:**

Taking into account both nucleotide interactions and the spacer length of p53-RE, we have created a novel *in-silico *regression-based transactivation capability predictor for p53-REs and used it to analyze validated and novel p53-REs and to predict the impact of SNPs overlapping these elements.

## Background

More than half of human cancers have a mutation in the tumor suppressor protein p53 or one of its target genes [1]. The p53 gene has been implicated as a master regulator of genomic stability, cell cycle, apoptosis, and DNA repair [2-5]. p53 regulates its target genes through binding specifically to a palindromic consensus sequence, RRRCWWGYYY-(spacer of 0–13 bp)-RRRCWWGYYY [6]. Since the consensus-binding site for p53 has been established [6], many p53 target genes have been identified experimentally [7-10]. Computational algorithms were also developed to explore the potential p53-response elements (p53-REs) on a genomic scale [10,11]. Currently, there are > 150 experimentally verified p53-RE sequences, with > 1500 high-probability p53 loci [11,12]. One feature of p53, however, confounds the discovery of novel transregulated genes; while some binding sites match the expected consensus sequence quite well, others can be consensus-poor and yet are both necessary, and sufficient, to transactivate a gene [13]. Not surprisingly, nearly all known REs are reported to contain at least one mismatch in the decamer [6,11]. A recent study noted that although the spacer region between half sites for p53-REs can range from zero to 13 bases, smaller spacer lengths are preferred [11,12].

Computational approaches for identifying putative p53-REs from the target genes are based on position weight matrices (PWMs). These PWMs are matrices with expectation frequency defined for each nucleotide at each position of the REs. Though commonly used, PWMs in general have their own limitations (see [14] for details), and two of these limitations are applicable to p53-REs: i) PWMs cannot define motifs of variable lengths, and ii) PWMs cannot model interactions between nucleotides. In the case of p53-REs, even though the two constituent half-site length is fixed (10 bp long), the RE length itself varies because of the variable length of the spacer separating the two half-sites. Additionally, the nucleotide interactions within the p53-RE define its binding affinity [9,15]. Building on these rudimentary profile scores, more sophisticated methods like p53MH have been developed [16]. However, these methods are based on REs known either to bind or not bind p53 and not on their activity and impact on p53 transactivation itself. In general, the degree of responsiveness depends on various factors including the state of the p53 protein [17], its cofactors [18], and the sequence composition of the p53-RE itself [19]. Although a recent prediction method takes into account experimentally derived protein saturation levels for various p53-REs mutated systematically [20], it does not take into account the spacer length or composition in p53-REs. Instead it considers the effect of individual nucleotides on binding affinity as additive.

Extending on an earlier methodology [21], in the current study, we developed a two-step procedure for quantitative prediction of the p53-RE transactivation capability. In the first step, we used multidimensional scaling to map all the training p53-REs into a Euclidean space. In the second step, we used multinomial logistic regression to regress the distance between the p53-REs in the Euclidean space against their known binding affinities. The training data for relative transactivation of p53-REs were obtained from our recent study [8], wherein, using a combination of custom bioinformatics and multispecies alignment of promoter regions, we investigated the functional evolution of p53-REs in terms of responsiveness to the p53. We identified REs orthologous to known p53 targets in human and rodent cells or, alternatively, REs related to the established p53 consensus. The orthologous REs were assigned p53 transactivation capabilities (in terms of "on" or "off" and level of response) based on rules determined from model systems [22]. The underlying hypothesis for the current study is that p53-REs with similar binding site composition and spacer length have similar transactivation capability. Our goal is to predict the transactivation score of a novel p53-RE based mostly on the dissimilarity or distance from existing known p53-REs with known transactivation capability. We demonstrate the utility of our model by (a) ranking putative p53 target genes based on their predicted transactivation; (b) comparing the performance of our approach with a previously reported method [20]; (c) identifying and ranking putative p53-target microRNA promoters; and (d) predicting the implications of single nucleotide polymorphisms (SNPs) within p53-REs on p53 transactivation.

## Results and discussion

### Regression-based transactivation capability predictor for p53

We used 353 previously validated p53-REs along with their transactivation capabilities from 14 different species [8] for training and testing a regression-based p53 binding predictor. Briefly, we used multidimensional scaling to map all the training p53-REs into a Euclidean space followed by multinomial logistic regression to regress the distance between the p53-REs in the Euclidean space against their known binding affinities. We used the distance between the validated p53-REs and their spacer lengths as features for training a multinomial logistic regression model (see Methods for additional details). Our method was based on a similar affinity predictor designed for NF-Kappa B [21]. However, contrary to NF-Kappa B, p53-REs are not of fixed length primarily because of the varied spacer lengths separating the two half-sites. Earlier publications [6,11] on p53-REs point out that the binding affinity of the RE depends on the sequence of the dimer and the length of the spacer. Hence, for training purposes we ignored the sequence of the spacer and formed 20-mer sequences from the training data. Overall there were 263 unique p53-REs having spacer lengths ranging from 0 to 13 bp.

**Figure 1:**
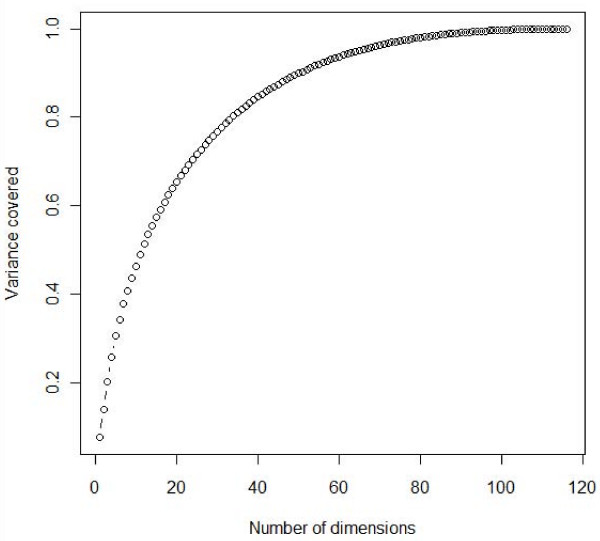
**Graph showing the variance of the model captured with respect to the number of input dimensions (Eigen values)**. At 50 dimensions, 90% of the variance or complexity of the model is captured.

We used multidimensional scaling [23] to project these 263 sequences onto a multidimensional Euclidean space such that the distance between any two sequences was approximately equal to their dissimilarity. We were able to transform these sequences into a 116-dimensional subspace. Though 90% of the variance in the data could be captured by just 50 dimensions, we decided to retain all the 116 dimensions for accuracy and also because these dimensions would be automatically obtained for a novel p53-RE. It is therefore reasonable to conclude that 50 dimensions capture the complex nucleotide interactions that are ignored by earlier additive models. Figure 1 shows the percentage of variance captured as a function of number of dimensions (see methods for calculating variance from number of dimensions). In addition to the Euclidean space dimensions, we also obtained the spacer associated with each 20-mer p53-RE in the training set. On the whole, we used 116 (Dimensions) + 1 (spacer) = 117 features as input to the regression analysis.

### Performance and usability of the model – Cross validation

**Figure 2:**
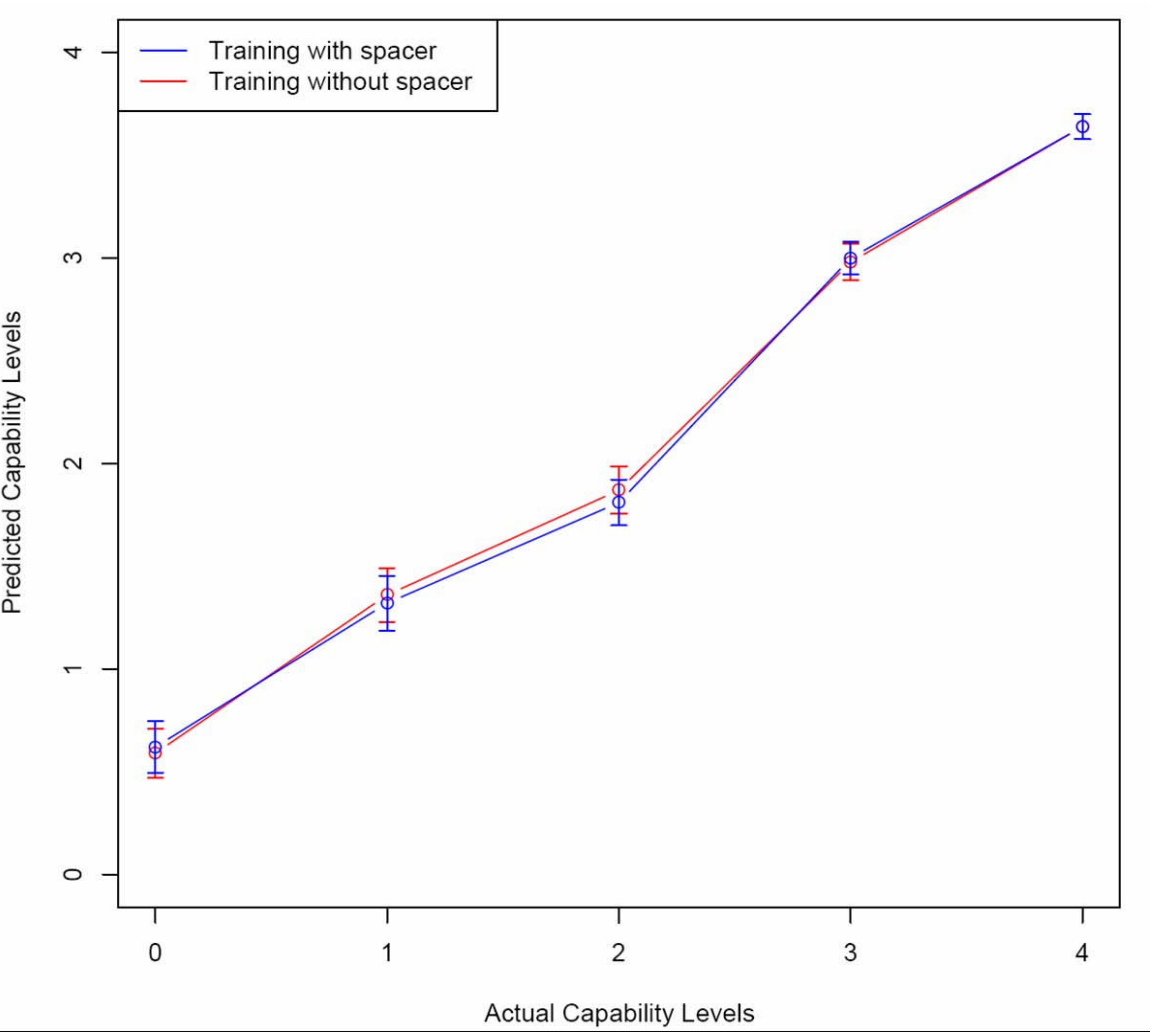
**Leave-one-out cross-validation results showing a straight line between the actual and observed transactivation capabilities**. The average predicted values for lower levels of transactivation do not exactly follow the observed levels.

We used ten-fold and leave-one-out cross validations to test the performance and usability of our model. Pearson correlation coefficients were calculated between observed and predicted transactivation capabilities. For ten-fold cross-validation we obtained correlations of 0.71 and 0.73 (0.71 ± 0.06 and 0.73 ± 0.05 respectively if correlation is calculated for each fold separately) for models without and with spacers, respectively. In the case of leave-one-out cross-validation, we obtained correlations of 0.71 and 0.70 for models without and with spacers, respectively. We were unable to find correlation for each fold separately as each has only one test case in leave-one-out cross-validation. Surprisingly, we did not observe a significant difference between training with and without spacers. This could probably be because the training data spacer distribution is highly skewed toward the lower values. In other words, only 12 of the 263 p53-REs had a spacer of length 8 bp or higher. Nevertheless, we noted some improvement in the performance (ten-fold cross-validation) when spacers was used as a feature, although it is not statistically significant. To test whether the correlation results are skewed toward a specific transactivation capability value, we obtained the average predicted capability for each level of true capability. Figure 2 shows the "predicted" and "observed" transactivation capabilities for leave-one-out cross-validation. Both the models – without and with spacers – performed similarly. However, toward the lower levels of observed capability, we noticed a slight increase in the average predicted capability levels, though not statistically significant. This was especially apparent for levels 0 and 1, which correspond to "Non-responsive" and "Poor" transactivation capabilities, respectively. Both models performed well in predicting the higher capability values.

In addition to the five different levels of binding, the model can also be used simply to test if a specific p53-RE could be functional or not. For this, we considered the capability levels "Non-responsive" and "Poor" to be non-functional, while the categories "Slight", "Moderate", and "High" were classified as functional. A leave-one-out cross-validation with this assumption resulted in a sensitivity of 0.84 and specificity of 0.79.

### Comparison with other methods

To compare the performance of Veprintsev's model [20] with our approach, we ran their algorithm on the same set of 263 REs we used for training. The correlation was only -0.23 between the predicted and observed output. Since a comparison between categorical observed transactivation capability and continuous predicted binding affinity is not really intuitive, we divided the input test set into functional and non-functional REs as described in the previous sections. We also divided the predicted affinity into functional and non-functional based on a default cut-off of -6.0 as provided by the software. We noticed that while the sensitivity of the predictor was a high 0.91, the specificity was only 0.27, suggesting that the predictor inaccurately overestimates a non-functional RE as functional about 63% of the time. On the contrary, this estimate was only 21% using our model. To further confirm this we divided the input test set as non-functional if capability level was "Non-responsive" and functional for other capability levels. Using Veprintsev's model, we obtained a sensitivity and specificity of 0.90 and 0.35, respectively, while for our model it was 0.90 and 0.66, respectively. Although we observed a moderately decreased specificity for our model, it is still better than the 0.5 cut-off for a random predictor. In spite of the high false positive rate the simplistic additive basics of the Veprintsev p53 algorithm make it a good complementary tool for affinity prediction.

### Transactivation capability prediction of known validated p53-REs

**Figure 3:**
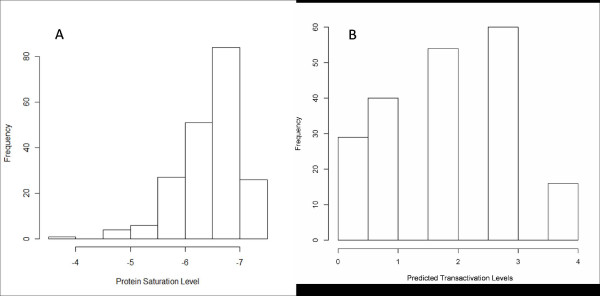
**(A) Frequency distribution of protein saturation level scores from Veprintsev's algorithm for detecting p53 RE binding affinity applied on validated p53 for detecti REs**. (B) Frequency distribution of categorical transactivation level prediction scores from our algorithm applied on validated p53 REs.

A total of 199 unique known validated human p53-REs of at least 20 bp length were obtained from four publications, namely, Jegga et al. [8], Horvath et al. [7], Riley et al. [10], and Ma et al. [9]. We obtained the predicted tranasactivation capability and binding affinity from our model and the algorithm from Veprintsev et al. [20], respectively. Figures 3A and 3B show the frequency distribution of the predictor output of Veprintsev and our model, respectively. The frequency distributions highlight two important aspects. First, most of the validated p53 binding sites are predicted positive by both of the algorithms, 80.9% by Veprintsev and 85.4% by our model. Second, the distributions follow normality skewed toward the higher binding affinity/transactivation capability levels. These results confirm the veracity of the algorithms and their conformity with each other in terms of sensitivity.

**Figure 4:**
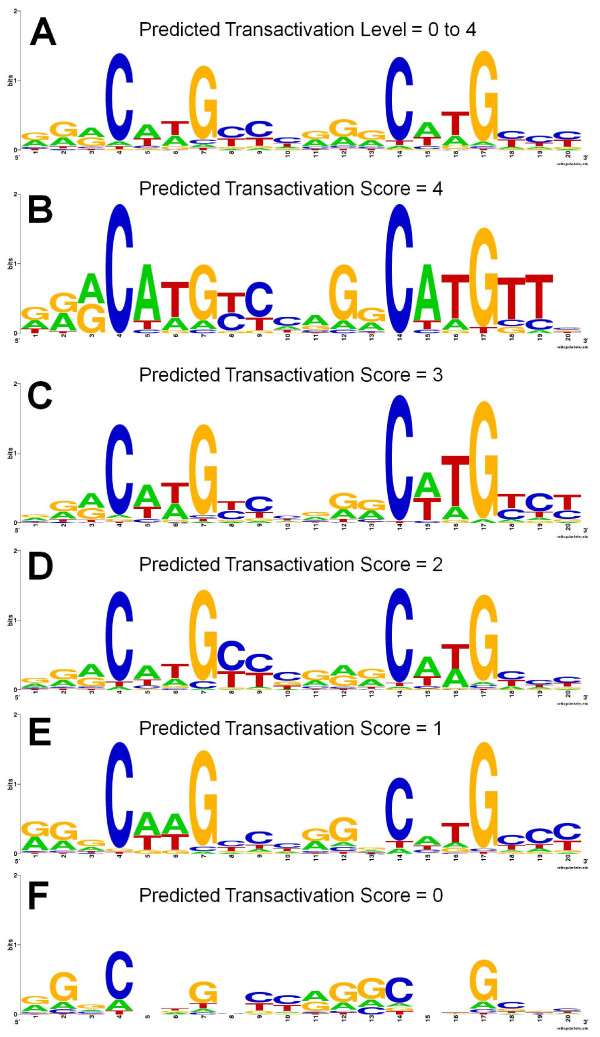
**Sequence logos separated by categorical transactivation prediction levels from our algorithm applied on validated p53 REs**. (A) Sequence logo formed using all validated p53 REs score. (B) Logo formed using predicted transactivation level of 4 (High), (C) using level 3 (Moderate), (D) using level 2 (Slight), (E) using level 1 (Poor), and (F) using level 0 (Non-responsive).

To further analyze the relationship between predicted transactivation capability levels obtained through our method and the validated p53-RE sequence features, we first separated the p53-REs by their capability levels. Using WebLogo [24] we obtained the consensus sequence logos representing the frequency of each nucleotide at each position for each of the capability levels (Figure 4). Not surprisingly, the consensus (sequence logo in Figure 4a) obtained by including REs corresponding to all capability levels revealed an enrichment of nucleotides ''C'' and ''G'' in the CWWG core of the p53-RE. This was in fact irrespective of all capability levels. Figure 4b shows the consensus logo formed by sequences including only p53-REs categorized under transactivation capability level of ''4''. We observed that several REs had ''AT'' in the CWWG consensus, including the lower capability categories. However, it is worth noting that the sequences with predicted transactivation level ''0'' (Figure 4f), had a weak CWWG consensus. Additionally, many purines (A/G) were observed in the "YYY" consensus of the second dimer. All these results highlight the differences between the predicted lower capability p53-REs and the p53 consensus.

**Figure 5:**
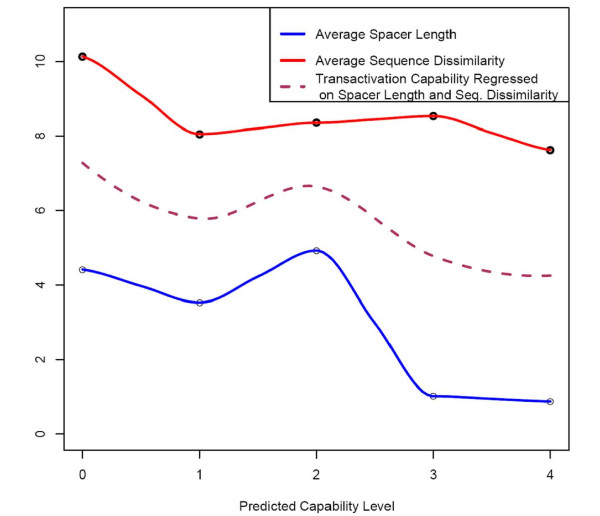
**Correspondence between predicted transactivation levels obtained from validated p53 REs with its dimer sequence dissimilarity and spacer lengths**. Dimer sequence dissimilarity is calculated as the distance from the best p53 RE, (GGGCATGCCC)_2_. Also shown is a local regression curve fit on dimer dissimilarity and spacer length. All the three measurements are in general negatively correlated to the predicted transactivation scores.

Since it is well known that the transactivation capability of the p53-RE depends both on the sequence composition of the dimers and the spacer length, we next analyzed the variation in the p53-RE sequence from the consensus, and the variation in the spacer length with respect to the transactivation capability (Figure 5). Both the average sequence dissimilarity and the spacer length showed a decreasing trend with respect to the transactivation capability. However, there was a slight increase in the average spacer length for capability value "2" compared to capability value "0." Considering that transactivation capability is affected by both dimer sequence dissimilarity from consensus and the spacer length, we fitted a curve on these variables. We noticed a distinct pattern wherein there was a decreasing pattern of the curve with increasing transactivation capability. However, we still noticed some deviation from the decreasing pattern. There could be several reasons for this: i) when measuring the p53-RE sequence dissimilarity, the consensus sequence was taken to be that with the highest transactivation capability (i.e., GGGCATGCCC)_2_); ii) previous studies reported a bimodal induction of transactivation capability, especially with spacer length [15]; and iii) several other features like interaction between nucleotides that are captured by our predictor could affect the transactivation capability to deviate from the expected value (See Additional File 1 for a complete set of predictions for each validated p53 binding site).

### Transactivation capability prediction of non-validated p53-REs

After initial testing that our algorithm is capable of predicting a significant number of validated p53-REs as functional, we sought to rank the known human p53-REs (not necessarily experimentally validated) reported in the literature based on their putative transactivation capability predicted by our approach. To do this, we compiled 2026 REs from the literature [11,25,26]. These literature-compiled p53-REs represent a collection of high-confidence putative p53 binding sites obtained using ChIP-Chip and *in-silico *methods. In order to further prioritize or rank these p53-REs based on their predicted transactivation capability, we used p53MH [16] to obtain the p53-RE scores and then applied our predictor. Although several of these p53-REs were predicted positive by both p53MH and our algorithm, only 23 of them had a p53MH score of 100 and a high capability score of "4" by our algorithm (Table 1) and of these only 3 p53-REs (of genes *PPM1J*, *DDB2 *and *PLK2*) have been experimentally validated. Additional file 2 shows the scores for all the known p53-REs (sorted by p53MH score and transactivation capability predictions).

**Table 1:** Twenty-three p53-REs predicted positive by both p53MH and our algorithm

**Gene**	**chr**	**p53-RE Start**	**p53-RE End**	**p53-RE_Dimer 1**	**p53-RE_Spacer**	**p53-RE_Dimer 2**	**Spacer Length**	**Ref.**
***PLK2***	**5**	**57793857**	**57793877**	**GGGCAAGTCC**		**AGGCATGTTT**	**0**	[11]
***PPM1J***	**1**	**113048061**	**113048081**	**GGGCTTGCTC**		**AGGCATGTTC**	**0**	[25]
***DDB2***	**11**	**47193105**	**47193126**	**GAACAAGCCC**	**T**	**GGGCATGTTT**	**1**	[11]
*KIAA1486*	2	226209743	226209763	GAACATGCCT		GGGCTAGCCT	0	[11]
*MTHFD1L*	6	151220175	151220195	GGACATGCCT		GGGCATGTCC	0	[11]
*PRKAG2*	7	151016282	151016302	GAGCATGTCT		GAACATGTTC	0	[11]
*AKAP6*	14	31884451	31884471	AGACATGTTT		GGGCATGTCT	0	[25]
*BIRC8*	19	58490331	58490351	GGACATGCCT		GGGCATGTCT	0	[25]
*APBB2*	4	40721763	40721784	AAACTTGTTT	C	AGGCTAGCCC	1	[26]
*TSHR*	14	80618516	80618537	AAACTTGCTT	C	AAGCTAGCCC	1	[25]
*DMD*	X	31592231	31592252	AAACATGCTC	T	GGACTAGCCT	1	[25]
*SLCO2B1*	11	74540123	74540146	GAGCAAGCCT	GGG	GGACATGTTC	3	[26]
*ATF3*	1	210865885	210865908	AGGCAAGTCC	TCA	GAGCATGTTT	3	[11]
*FRMD4A*	10	14167552	14167577	AAGCTTGCTT	TCAGA	GGGCTTGCCT	5	[11]
*EGFR*	7	55176461	55176487	AAACATGCCT	TTCAAA	GAACTAGTTC	6	[25]
*MMP2*	16	54067700	54067729	AGGCAAGTCC	ATAAAGTGA	AAGCAAGTTT	9	[11]
*KRT15*	17	36930241	36930270	GAACATGCCC	TGTGAGCCT	GAGCATGTTC	9	[25]
*DLG2*	11	83032605	83032636	GAACATGTCC	ATGGCTGTCTC	AGACTTGTTT	11	[25]
*NRXN3*	14	78316130	78316162	AGACTTGCCC	AACTAGACATCA	AGGCATGTTT	12	[25]
*FHIT*	3	61206990	61207024	AAACTTGCTT	TCACTTTACTCTGT	GGACTTGCCC	14	[26]
*DOCK9*	13	98270727	98270761	GGGCAAGTCC	ACAGTGCAAAGTAA	AAGCAAGTTT	14	[25]
*GRIN2A*	16	9796860	9796894	AAACTTGCTT	TGACTTTACTCCAT	GGACTTGCCC	14	[25]
*ACCN1*	17	28722009	28722043	AGGCAAGTCC	GCAGTGCAAAGCGA	AAGCAAGTTT	14	[25]

These 23 top ranked REs had a p53MH score of 100 and a transactivation capability score of "4" by our algorithm. Of these, only 3 p53-REs (of genes *PPM1J*, *DDB2 *and *PLK2*; bold font) have been experimentally validated in previous studies.

### Prediction of transactivation capability for putative p53-REs in microRNA promoters

We used the "high confidence" microRNA promoters (59 promoters directing transcription of 79 microRNAs) from Fujita and Iba [27] and in the first step ran the p53MH algorithm [16] to obtain putative high-scoring p53 binding sites in these miRNA promoter regions. The p53MH parameters were set to obtain only the top 3 high-scoring p53-RE matches. In the second step, we used our transactivation scoring model to predict the transactivation capability of each of the p53-RE matches. Out of 180 putative p53-REs occurring in 60 microRNAs, 51 p53-REs (corresponding to 30 microRNAs) were predicted with high scores (> 70) by p53MH. Out of these 40 REs (25 microRNAs) were predicted with a transactivation score of at least "1" by our model. We intersected our results with a list of miRNAs that have been reported to be either induced or suppressed following p53-activation [28]. We found that 6 induced (mir-106a, mir-128a, mir-191, mir-21, miPPR-23b, and mir-34a), and 3 repressed (mir-671, mir-125b, and mir-100) microRNAs had high-scoring putative p53-REs (see Additional file 3). The fact that mir-34a, the known p53-regulated miRNA, was identified by our model as a high affinity target (apart from a p53MH score of 100) supports the ability of our model's potential in predicting p53-REs' transactivation capability.

### Performance of regression model with varying spacer length

Although variable p53-RE spacer lengths are known to affect transactivation capacity [29], to the best of our knowledge none of the current algorithms consider spacers as one of the parameters when predicting the transactivation capability of p53-RE. Thus, for the first time, we have incorporated spacer length as one of the features in our regression model for predicting the transactivation capability of p53-RE. To test specifically the performance of our model in predicting the transactivation capability of REs with different spacer lengths, we compiled literature-reported validated p53-REs and artificially varied their spacer length (from 0 to 14 bp), keeping the dimer composition constant. For simplicity, we have grouped the results into different categories based on the fold-change (4-fold, 3-fold, 2-fold or 1-fold) in transactivation capacity with varied spacer lengths. For example, a change in transactivation score from "4" to "0" with an increase in spacer length corresponds to a 4-fold change.

#### Weak p53-RE half-sites show increased transactivation capability when spacer length is reduced

**Figure 6:**
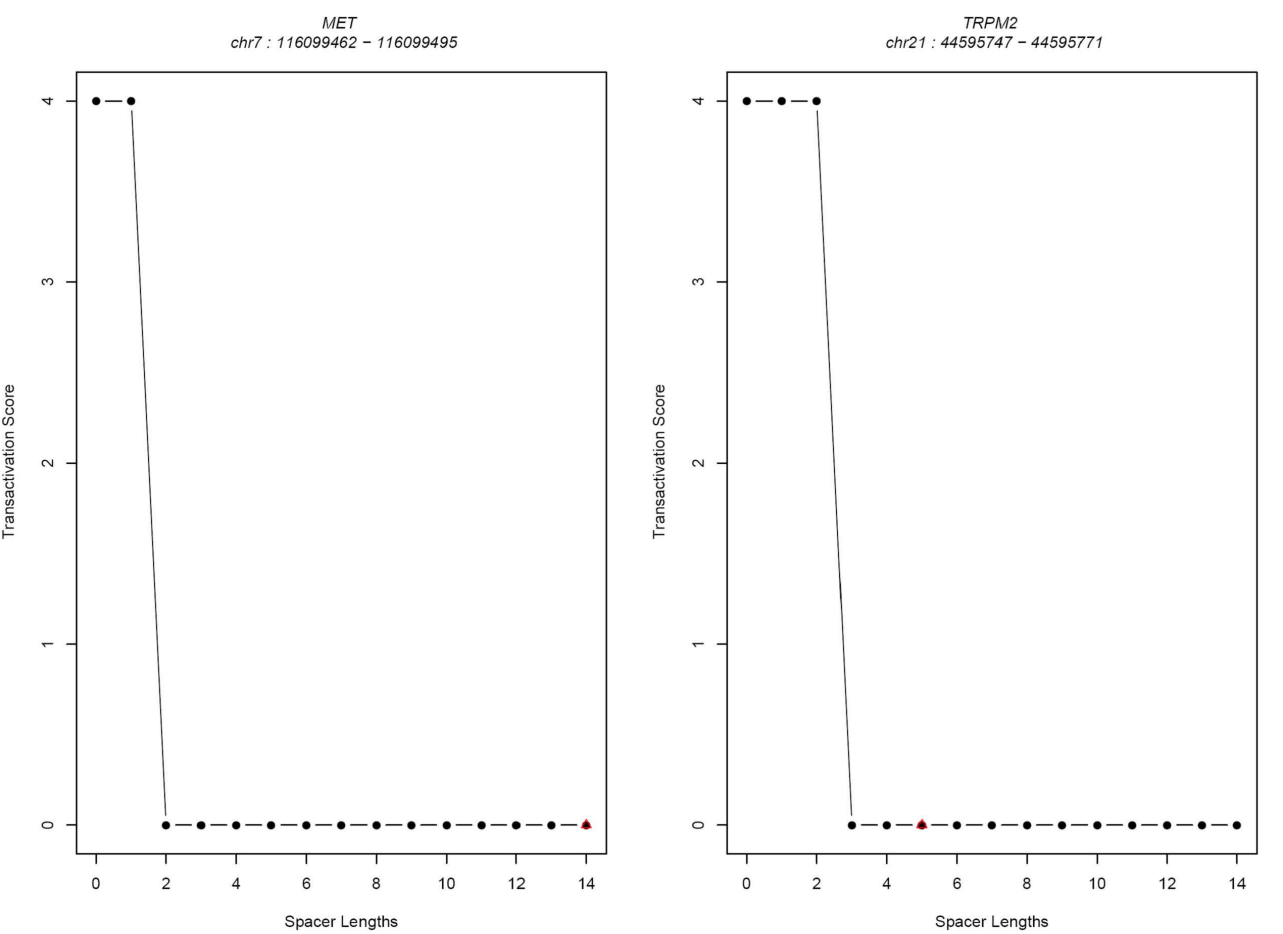
**Four-fold change in predicted transactivation capability of validated p53 REs with varying spacer lengths**.

**Figure 7:**
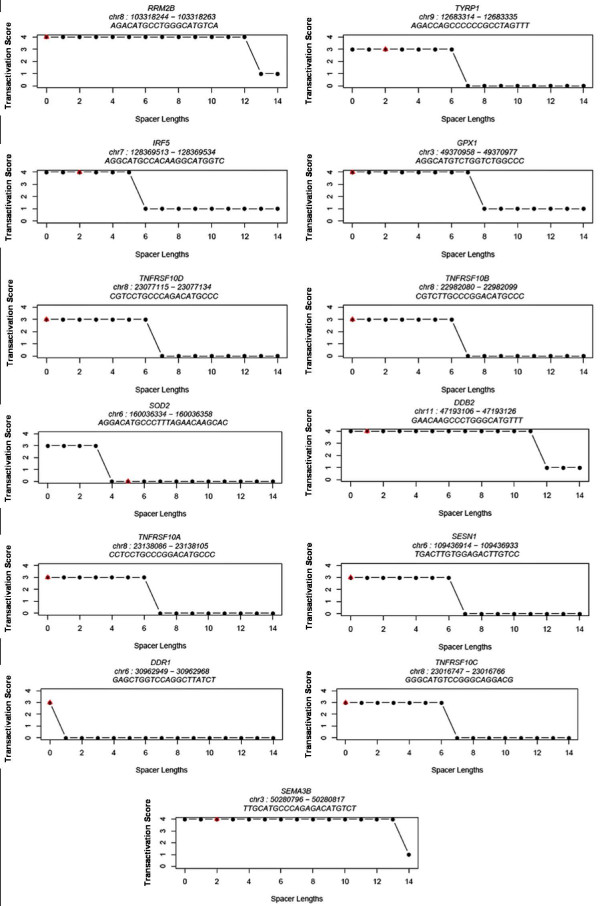
**3-fold change in predicted transactivation capability of validated p53 REs with varying spacer lengths**.

In order to test whether a lower spacer length would increase the predicted capacity of the REs with weaker dimers, we tested all possible spacer lengths from 0 to 14 bp (see Methods). We selected two validated p53 target genes (*MET *and *TRPM2*) with a 4-fold change difference when the spacer length was artificially varied for the analysis of implication of spacer length in p53-RE transactivation capacity. While the functional p53-RE of *MET *not only has mismatches in the core CWWG but also has a spacer of 14 bp (GGACGGACAG-14 bp spacer-AGACACGTGC), *TRPM2 *p53-RE (GGCCTTGCCT-5 bp spacer-AGGCCTGCTT) has a spacer length of 5 bp. Interestingly, *MET *p53-RE was predicted to have an increased transactivation capability (4-high) if the spacer length were artificially reduced to 0 or 1 bp (Figure 6). Likewise, *TRPM2 *p53-RE was predicted to have a capacity of 4 (high) if the spacer lengths were lower (i.e. 0, 1, or 2 bp). An alternate example is p53-RE of *DDR1 *(GAGCTGGTCC-0 spacer-AGGCTTATCT) (Figure 7), whose predicted transactivation score drops to zero when the spacer length is increased by 1 bp! In a recent systematic analysis measuring the ability of the p53 to transactivate 1/2 site or 3/4 sites [30], it has been suggested that two weak half-sites may actually be a functional 3/4 site.

#### Strong p53-RE half-sites retain high transactivation capability irrespective of spacer length

Since p53-REs that are in strong agreement with the consensus are known to have higher transactivation capability [9,29], we selected those REs that have a high similarity to the consensus (especially in the core *CWWG*) and predicted the effects of spacer length on their transactivation by varying the spacer length (0–14 bp). For instance, the functional p53-RE of *RRM2B *(chr8:103318244–103318263) has *CATG *in both the dimers (Figure 7), and we found that increasing the spacer length does not alter the transactivation significantly. Similar results were obtained for target genes *DDB2 *and *SEMA3B *(Figure 7). These results are in complete agreement with earlier findings that the effect of spacer is partially overcome by the presence of a strong *CWWG *core in the dimers [31]. List of p53 REs with 1-fold and 2-fold change in transactivation scores with varying spacer lengths are included as additional files (Additional Files 4, 5, and 6).

### Implication of SNPs on p53-RE transactivation capability

Although several computational approaches exist to predict the impact of coding and non-coding polymorphisms [32], very few take into account the binding affinity of a transcription factor with the response element, let alone predict their impact.

#### Effect of SNPs overlapping p53-RE half-sites

Using the p53-REs as a test case, we sought to assess the impact of human non-coding single nucleotide polymorphisms (SNPs) on the p53-RE transactivation capability. To do this, using the UCSC genome browser [33], we made an intersection of 199 validated p53-REs and human non-coding SNPs. There were 36 non-coding SNPs overlapping with a known validated p53-RE (Table 2; see also Additional files 7 and 9 for a complete list of validated p53-RE overlapping SNPs along with the predictions of their effects on transactivation). Of these, 33 overlapped with dimers, out of which 10 SNPs were predicted to impact the transactivation capacity by our predictor. For instance, a G>C variation (rs2228108) in the *TAP1 *gene (occurring at +643 bp from TSS), decreased the predicted transactivation score from "3" to "1." The variation alters the "G" of the core motif CWWG in the first dimer to "C" which could result in reduced transactivation capability. A similar result (9-fold change in the binding affinity) was obtained when we repeated the analysis using Veprintsev's algorithm [20]. Likewise, a C>G variation (rs934345) occurring upstream to *DCC1*, and overlapping a validated p53-RE, is predicted to increase the transactivation capability from "2" to "3." The *DCC1 *p53-RE has "CAG" for the "RRR" in dimer1 (native RE), which changes to "GAG" because of a SNP (C->G) and could be responsible for increasing the predicted transactivation score. Thus, our algorithm is not only sensitive to predict the implications following variations in the core "CWWG" but also to those occurring in the flanking sequences. However, there were some exceptions – for instance, a SNP (rs702720; T->C) that overlaps the third purine in the RR*R *of the second dimer of a validated p53-RE. Although both the wild-type and minor allele are mismatches to the original p53 consensus, our model predicts an increase in the transactivation score from "1" to "2". This could be because of lack of sufficient training data that gives sufficient coverage throughout the entire variation space of the p53 consensus. Also, as discussed earlier there were only 12 p53-REs in the training set with spacer lengths greater than 8.

**Table 2:** Thirty-six non-coding SNPs overlapping with a validated p53-RE.

**Genes**	**p53 RE Location**	**Spacer Length**	**SNP ID**	**Allele**	**Wt-Binding Prediction**	**Minor Allele-Binding Prediction**	**Reference**
*SERTAD1*	chr19: 45623874–45623893	0	rs268682	C/G	0	1	[7]
*TAP1*	chr6: 32929058–32929083	6	rs2228108	C/G	3	1	[10]
*TP73*	chr1: 3597020–3597050	11	rs12121865	A/G	2	3	[8,10]
*PLK2*	chr5: 57793125–57793147	3	rs702720	A/G	1	2	[7,9,10]
*HSP90AB1*	chr6: 44322842–44322871	10	rs35074133	A/T	2	3	[10]
*BDKRB2*	chr14: 95740864–95740883	0	rs1800508	C/T	0	3	[9,10]
*DSCC1*	chr18: 48118859–48118878	0	rs934345	C/G	2	3	[7]
*TP73*	chr1: 3556376–3556406	11	rs12040834	G/T	1	2	[10]
*EEF1A1*	chr6: 74286408–74286431	4	rs11550799	G/T	1	2	[9,10]
*EEF1A1*	chr6: 74286408–74286431	4	rs11550790	G/T	1	2	[9,10]
*EEF1A1*	chr6: 74286408–74286431	4	rs11556652	C/T	1	3	[9,10]
*EEF1A1*	chr6: 74285585–74285606	2	rs11556679	C/G	1	1	[10]
*EEF1A1*	chr6: 74286408–74286431	4	rs11550844	C/T	1	1	[9,10]
*KRT8*	chr12: 51585038–51585059	2	rs11554493	C/T	0	0	[10]
*EEF1A1*	chr6: 74285784–74285805	2	rs11556702	A/G	2	2	[10]
*ADARB1*	chr21: 45316682–45316701	0	rs2838769	A/G	2	2	[10]
*SCGB1D2*	chr11: 61765841–61765860	0	rs2232945	A/G	3	3	[7]
*TP63*	chr3: 190989527–190989549	3	rs9844460	C/T	4	4	[10]
*EOMES*	chr3: 27739623–27739643	1	rs3806624	C/T	2	2	[7]
*PMS2*	chr7: 6012202–6012223	2	rs2881029	A/C	1	1	[7,10]
*SIVA*	chr14: 103984243–103984262	0	rs11628179	G/T	3	3	[7]
*KRT8*	chr12: 51585038–51585059	2	rs13098	A/T	0	0	[10]
*PLK3*	chr1: 45038183–45038202	0	rs17880745	A/T	2	2	[8]
*PLK3*	chr1: 45038183–45038208	6	rs17880745	A/T	2	2	[7,10]
*HSPA8*	chr11: 122437379–122437406	8	rs11823704	A/C	0	0	[9,10]
*ARHGEF7*	chr13: 110602821–110602840	0	rs1658728	G/T	3	3	[7]
*RRM2B*	chr8: 103318244–103318263	0	rs28999675	C/G	4	4	[7-10]
*TP73*	chr1: 3556358–3556385	8	rs12040834	G/T	2	2	[10]
*TP73*	chr1: 3597020–3597039	0	rs12121865	A/G	3	3	[7]
*EEF1A1*	chr6: 74285784–74285805	2	rs11550818	C/T	2	2	[10]
*TRIM22*	chr11: 5668357–5668376	0	rs35926783	A/G	4	4	[10]
*EDN2*	chr1: 41720668–41720687	0	rs11572355	A/G	3	3	[7,8,10]
*CASP1*	chr11: 104411147–104411166	0	rs3809024	A/G	3	3	[7,10]
*HSPA8*	chr11: 122437379–122437406	8	rs41302367	A/G	On Spacer	On Spacer	[9,10]
*SLC38A2*	chr12: 45037706–45037735	10	rs7960147	C/T	On Spacer	On Spacer	[9,10]
*MSH2*	chr2: 47483388–47483420	13	rs1863332	A/C	On Spacer	On Spacer	[10]

Of these, 33 SNPs overlap with dimers, out of which 10 SNPs were predicted to impact the transactivation capacity by our predictor (See Additional File 9 for more details).

#### Effect of indels overlapping p53-RE spacer region

For analyzing the effect of indels overlapping spacers on p53 REs transactivation capability, we used Galaxy [34] to obtain the 17-species multiple alignments for both validated p53 REs. We then used a custom bioinformatics program to assess the level of conservation between species in the two dimers and the spacer separately. Indels occurring in the dimer and spacer were noted. We then ran the transactivation capability prediction algorithm on the p53 REs of each species. This way the level of sequence conservation and transactivation capability between species could be obtained. The algorithm was able to successfully predict differences in transactivation capability. For example, a validated p53 RE occurring on exon 4 of the *EEF1A1 *is highly conserved across multiple species. However, subtle differences exist. For instance, the human p53-RE has a dimer1+dimer2 sequence of "GGGCATGCTCG**G**GT**C**TGCCC" and has a transactivation score of "1". But the corresponding frog sequence has a p53 RE that has a 20-mer sequence of "GGGCATGCTCG**A**GT**T**TGTCC" and has a transactivation score of "2." A C>T in the first "W" of the "CWWG" sequence in dimer2 results in the transactivation capability increasing by a unit of 1.

We also analyzed those p53 REs that have insertions or deletions in their spacers among the conserved species and predicted their transactivation capability. For example, a validated p53 RE in the 5'UTR of *BCL6 *has a spacer of length 13 and a predicted transactivation score of "1" in the human. When compared to other species, dog has a conserved p53 RE with a spacer length of 11 and a predicted transactivation score of "2" (see Additional file 8 for all of the multi-species alignments and predicted scores for validated p53 REs).

## Conclusion

Our p53-RE transactivation predictor is a useful complementary tool to current algorithms that are based on position-weight-matrices and experimental-based affinity values. Through various analyses we have shown that our method performs better than an existing algorithm by Veprintsev. We have done initial validation of our method by analyzing known validated p53-REs. We have shown the utility of this method as a valuable aid to the existing p53MH algorithm in obtaining high quality novel p53-REs. The results indicate that our model can predict the changes in the level of transactivation capability relative to changes in the spacer length. Additionally, our results corroborate the current theories on variation of binding affinities relative to spacer lengths. Based on our results we hypothesize that a deletion in the spacer (leading to smaller or no spacer) of a low-affinity RE could increase its transactivation capability while p53-REs with conserved consensus and high transactivation capability are tolerant of longer spacer lengths. We strongly believe that our method will help in prioritizing novel p53-REs obtained through various methods including high-throughput ChIP-chip experiments. Lastly, as more p53-RE transactivation experimental data becomes available, we anticipate an increase in the accuracy of our model.

## Methods

### Regression model for p53-RE transactivation capability

Our analysis is based on methods explained previously in Udalova et al. [21]. The known p53-REs with transactivation capability were extracted from Jegga et al. [8], and the pair-wise distance between each p53-RE was calculated as follows:

*d*_*ij *_min(*h*(*RE*_*i*_, *RE*_*j*_), *h*(*REi*, )), where *d*_*ij *_is the distance between i^th ^RE (*RE*_*i*_), and jth RE (*RE*_*j*_)

 is the reverse complement of *RE*_*j*_

*h*(*RE*_*i*_, *RE*_*j*_) is the Hamming distance between i^th ^and the j^th ^RE

A total of 263 unique REs were obtained from Jegga et al. [8] and hence the distance matrix ***D ***is of dimension 263 × 263. Let *n *denote this unique number of REs. We used the "cmdscale" function from "stats" package in R [35] for scaling the distance matrix to an (*n*-1) dimensional Euclidean space. We obtained the *m*-valid principal coordinates (Eigen vectors) from the output. When scaling, the pair-wise distance *d*_*ij*_, calculated earlier, is approximately equal to the Euclidean distance between the two sequences in the *m*-dimensional space. Thus, there were a total of 116 valid dimensions.

The consensus for p53 is two half-sites (10 bp each) of RRRCWWGYYYY separated by a spacer. We used the *m *valid principal coordinates and the spacer as features to train a multinomial logistic regression. In the training data there were 5 levels of transactivation scores ranging from 0 to 4. If there are Z levels the probability of observing a transactivation capability of level z in sequence i is given by



and



where 

**Figure 8:**
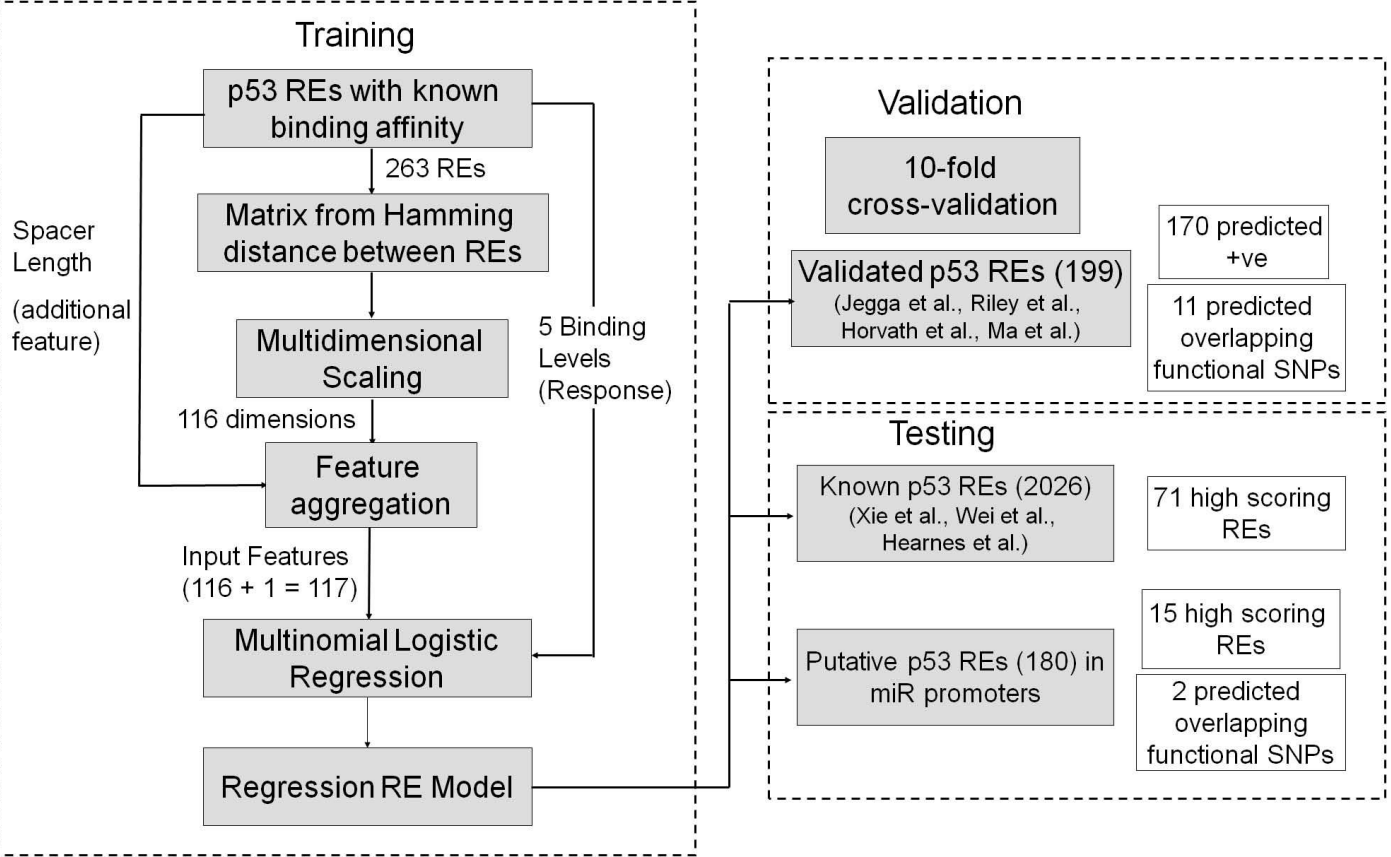
**Flow chart for training, validating, and testing logistic regression model for p53 RE transactivation capability prediction**.

In the above equations *α*_z _refers to the intercept for transactivation level *z*. **β **is a vector of regression coefficients. *x*_*ik *_refers to the *k*^th ^principal coordinate of the *i*^th ^sequence. *S*_*i *_refers to the spacer length of the *i*^th ^sequence. If an unknown sequence, not present in the training set, is the input, it is mapped into the Euclidean space using a kernel density function. This function is defined in Udalova et al. [21]. The transactivation level with the highest predicted probability is assigned as the predicted transactivation capability of the novel input sequence (see Figure 8 for the schematic representation of the methods).

### Variance and Eigen Values (Dimensions)

The variance of the original data captured by the first *n *Eigen values (here dimensions) can be defined as



where *ε *is a vector of all Eigen values and *N *is the total number of valid Eigen values.

### Correlation between observed and predicted affinities

If **a **is a vector of actual binding affinities and **p **is a vector of predicted binding affinities, the Pearson correlation coefficient between actual and predicted binding affinities is given by



where *n *is the total number of REs for which affinities are obtained.

### Calculating sensitivity and specificity of prediction algorithms





where TP = True positive predictions

   FN = False negative predictions

   TN = True negative predictions

   FP = False positive predictions

### Compiling validated and known p53-REs

Validated human p53-REs were compiled from literature (Jegga et al. [8] – 43 REs; Horvath et al. [7] – 83 REs; Riley et al. [10] – 151 REs; and Ma et al. [9] – 63 REs; of these, the last two [9,10] themselves are compilations of validated p53 REs from the literature). The p53-RE sequences of the compiled list were downloaded using BLAT and the UCSC table browser [33]. Similarly, known p53-REs (not necessarily experimentally validated) were also compiled from literature (Xie et al. [25] – 1196 REs; Wei et al. [11] – 428 REs; and Hearnes et al. [26] – 631 REs). Since the putative p53 target genes from Wei et al. and Hearnes et al. are based on genome-wide p53 binding maps using ChIP experiments, the exact position and the sequence of the p53 binding sites were unknown. The results in the publications are given in the form of p53 locus regions of length between 1 kb and 2 kb. We therefore ran the p53MH [16] algorithm on all the sequences obtained from the p53 binding loci. We set the threshold at 70 and restricted the output to three binding sites with the highest scores. Xie et al. scanned the -2 kb to +2 kb region of the human genomic transcription start site and scanned for motifs that are conserved at least across human, mouse, rat, and dog. In the MSigDB database [36], which is based on Xie et al., only the associated gene harboring the p53 binding sites is given. Hence, we scanned the -2 kb to +2 kb region of the genes from the database using p53MH with a cut-off score of 70 and restricted the output to the top three binding site matches.

### Spacer Analysis

For performing the spacer analysis we obtained all the validated p53 REs (199) as described earlier. After eliminating those REs with spacer length more than 14 we obtained 196 REs. Using a *JAVA *script, we constructed multiple entries for each REs with spacer length varying from 0 to 14 (keeping the half-sites constant) and noted the spacer length in the native RE. For each of these REs we then calculated the predicted transactivation capability through our regression model. The graphical representations of the transactivation capability variations with spacer length were generated using the R-package.

### Overlapping SNPs for validated and putative microRNA REs

Using the custom track feature in the UCSC Genome Browser, we intersected the p53-REs' positional coordinates with human SNP ("snp128" – corresponding to NCBI's dbSNP 128) coordinates and downloaded all the SNPs intersecting with p53-REs. Using custom programs written in *JAVA *we found the precise location of the SNPs on the RE and classified them as those occurring within the dimer (or the half-sites) or the spacer. We used the UCSC table browser to get the annotations for SNPs such as the minor and wild-type (wt) alleles and the strand. We used this to create the altered sequence (replacing the affected base pair) and finally predicted the binding affinities for the native RE and the mutated RE (with the polymorphic base pair) separately and estimated the difference.

## Availability of the software

The transactivation predictor software is available upon request from the authors.

## Authors' contributions

SG and AJ conceived the study design, which was coordinated by AJ. SG designed and implemented the p53-RE transactivation-based ranking algorithm and along with AJ participated in the analysis and interpretation of results. SG and AJ drafted the manuscript. Both the authors have read and approved the final manuscript.

## Supplementary Material

Additional file 1 **A list of 199 validated p53 RE acquired from 4 publications with their transactivation prediction by our algorithm and binding affinity prediction by Veprintsev algorithm.** Click here for file

Additional file 2 **A prioritized list of 2026 predicted p53 RE acquired from 3 publications with their prediction score from p53MH and our algorithm.** Click here for file

Additional file 3 **A list of prioritized putative p53 REs that occur in high-confidence putative microRNA promoters based on scores from p53MH and our prediction algorithm are included.** Click here for file

Additional file 4 **A list of p53 REs with 1-fold change in transactivation capacity with varying spacer lengths (Part 1).** Click here for file

Additional file 5 **A list of p53 REs with 1-fold change in transactivation capacity with varying spacer lengths (Part 2).** Click here for file

Additional file 6 **A list of p53 REs with 2-fold change in transactivation capacity with varying spacer lengths.** Click here for file

Additional file 7 **A complete list of prioritized SNPs overlapping validated p53 REs with their annotations and predictions through our algorithm.** Click here for file

Additional file 8 **A complete list of inter-species polymorphisms occurring in the validated p53 REs along with their binding predictions through our algorithm.** Click here for file

Additional file 9 **A complete list of thirty-six non-coding SNPs overlapping with a validated p53-RE.** Click here for file
