# Supplementary figures and images for: Regression based predictor for p53 transactivation
Source: BMC Bioinformatics. 2009 Jul 14;10:215. doi: 10.1186/1471-2105-10-215 (PMC2719629; doi:10.1186/1471-2105-10-215)

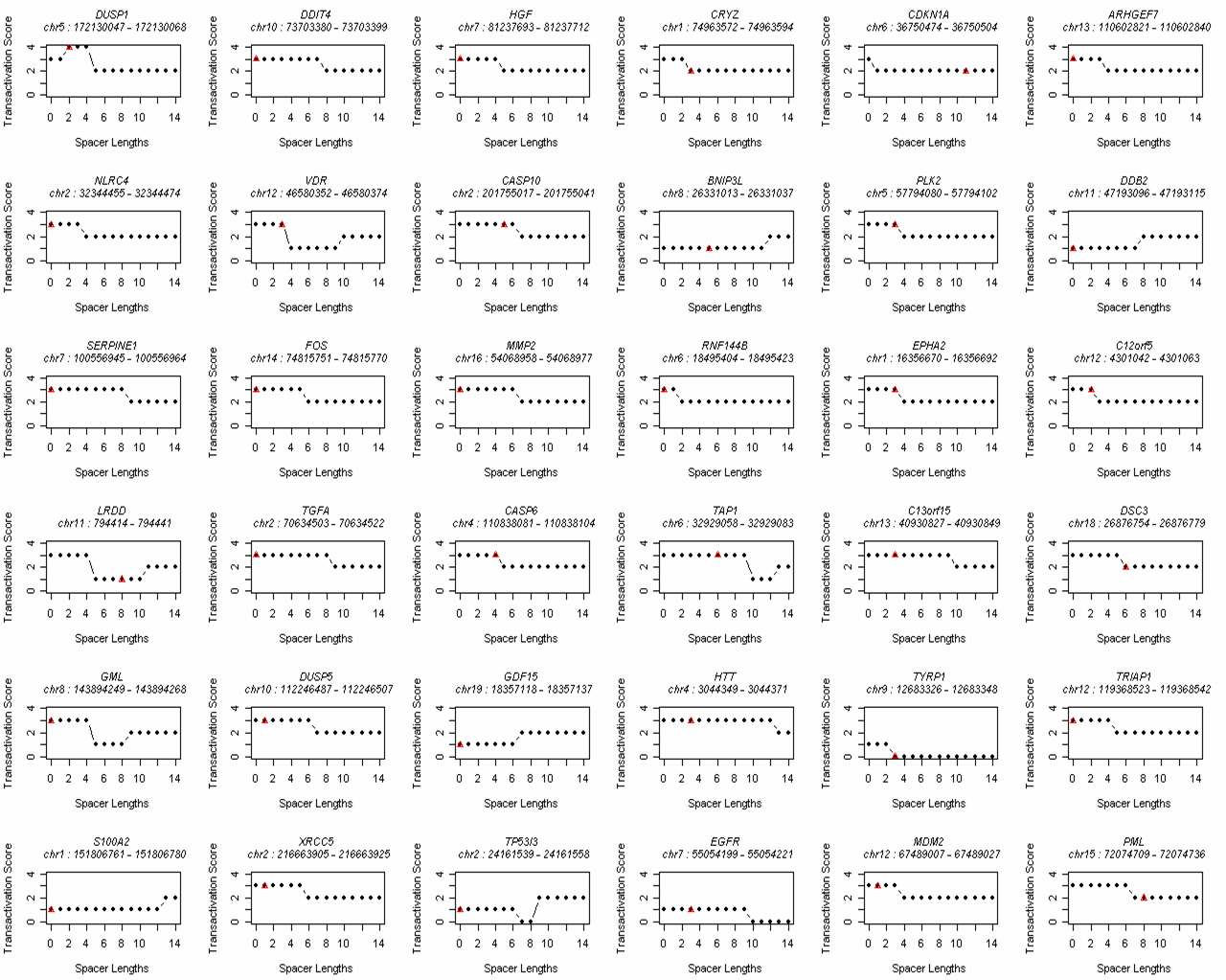

Supplement: Additional file 4 — A list of p53 REs with 1-fold change in transactivation capacity with varying spacer lengths (Part 1). [file 1471-2105-10-215-S4.tiff]

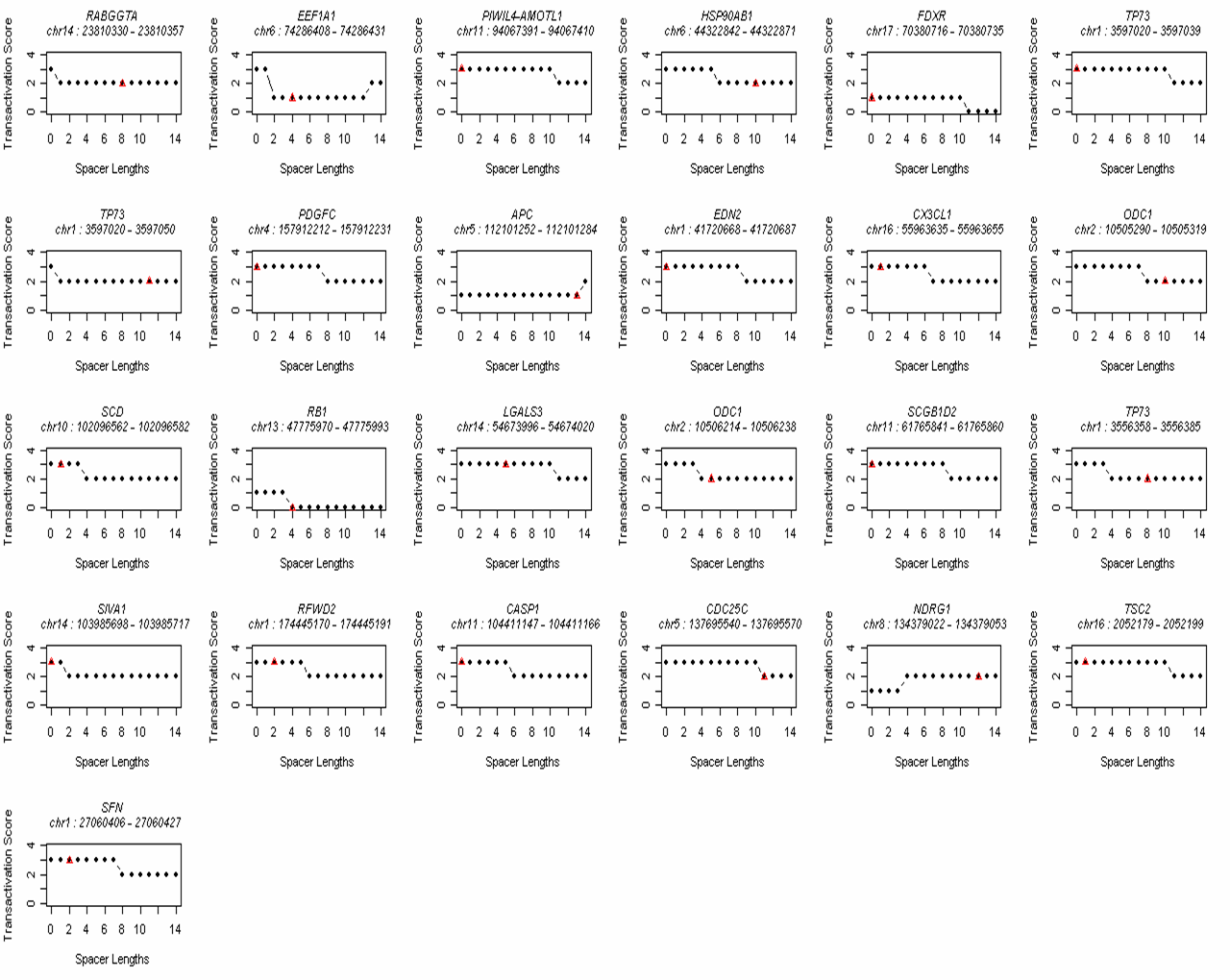

Supplement: Additional file 5 — A list of p53 REs with 1-fold change in transactivation capacity with varying spacer lengths (Part 2). [file 1471-2105-10-215-S5.tiff]

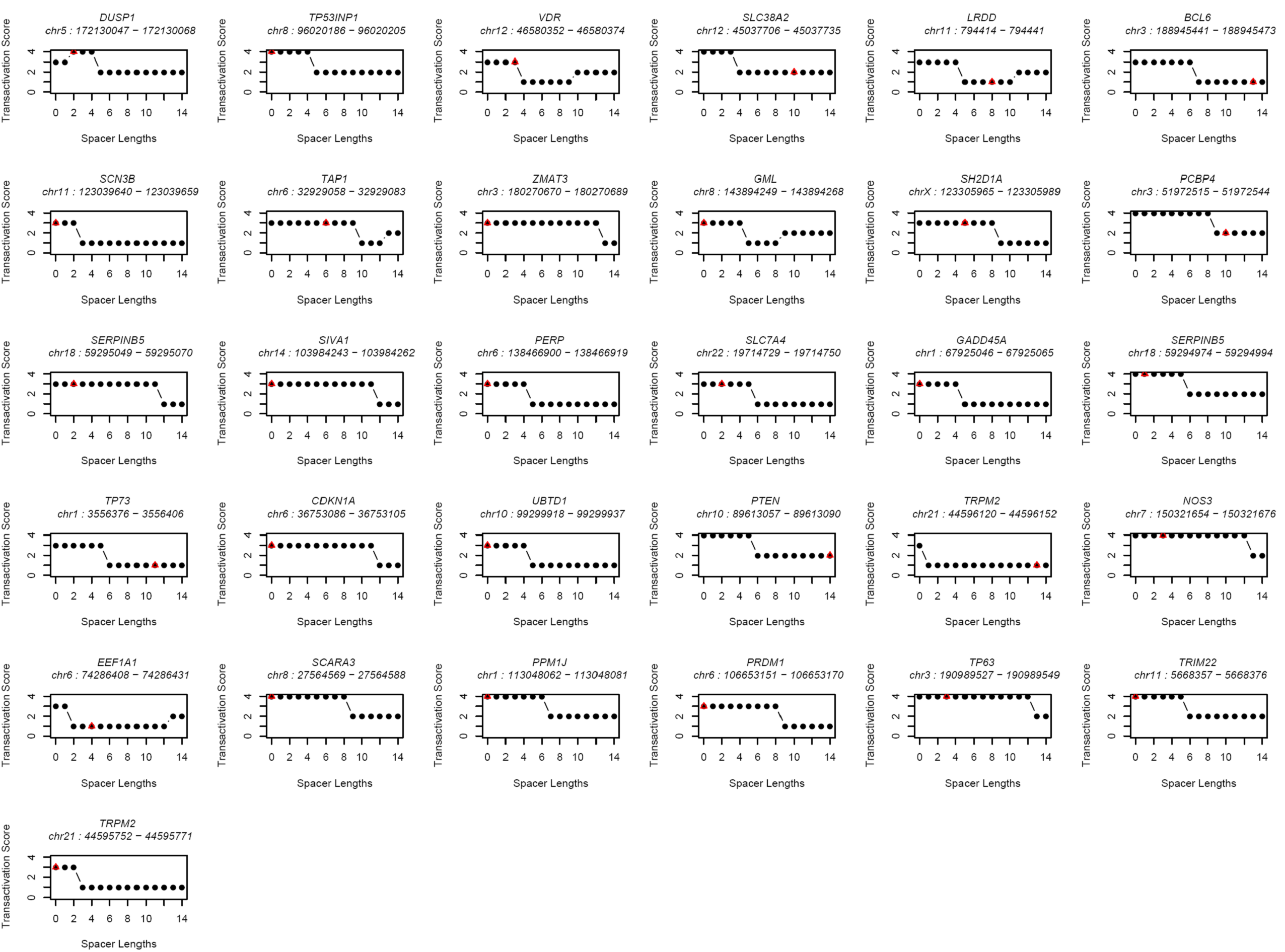

Supplement: Additional file 6 — A list of p53 REs with 2-fold change in transactivation capacity with varying spacer lengths. [file 1471-2105-10-215-S6.tiff]
